# Unsupervised Domain Adaptation for Inter-Session Re-Calibration of Ultrasound-Based HMIs

**DOI:** 10.3390/s24155043

**Published:** 2024-08-04

**Authors:** Antonios Lykourinas, Xavier Rottenberg, Francky Catthoor, Athanassios Skodras

**Affiliations:** 1 Department of Electrical and Computer Engineering, University of Patras, 26504 Patras, Greece; alykourinas@ac.upatras.gr; 2 Imec, 3001 Leuven, Belgium; francky.catthoor@imec.be (F.C.); xavier.rottenberg@imec.be (X.R.)

**Keywords:** Human–Machine Interfaces, ultrasound, hand-gesture recognition

## Abstract

Human–Machine Interfaces (HMIs) have gained popularity as they allow for an effortless and natural interaction between the user and the machine by processing information gathered from a single or multiple sensing modalities and transcribing user intentions to the desired actions. Their operability depends on frequent periodic re-calibration using newly acquired data due to their adaptation needs in dynamic environments, where test–time data continuously change in unforeseen ways, a cause that significantly contributes to their abandonment and remains unexplored by the Ultrasound-based (US-based) HMI community. In this work, we conduct a thorough investigation of Unsupervised Domain Adaptation (UDA) algorithms for the re-calibration of US-based HMIs during within-day sessions, which utilize unlabeled data for re-calibration. Our experimentation led us to the proposal of a CNN-based architecture for simultaneous wrist rotation angle and finger gesture prediction that achieves comparable performance with the state-of-the-art while featuring 87.92% less trainable parameters. According to our findings, DANN (a Domain-Adversarial training algorithm), with proper initialization, offers an average 24.99% classification accuracy performance enhancement when compared to no re-calibration setting. However, our results suggest that in cases where the experimental setup and the UDA configuration may differ, observed enhancements would be rather small or even unnoticeable.

## 1. Introduction

In recent years, Human–Machine Interfaces (HMI) have been introduced into numerous applications, including prosthesis control, robotic arm control, exoskeletons, smart wheelchair control, smart environment control, and exergaming [1]. The goal of an HMI is to transcribe user movements or movements intention into the desired action, thus allowing for an effortless and natural interaction between the user and the machine. Therefore, several types of sensing modalities, both of invasive and non-invasive natures, have been developed for monitoring user’s specific activities, such as hand, eye, limb, and joint movements. These types of sensing modalities measure different kinds of physiological signals that can be classified into three main categories: bio-potentials, muscle mechanical motion, and body-motion signals [2]. In order to accomplish their goal, HMIs leverage a wide variety of algorithms, ranging from simple thresholding to complex machine learning algorithms, for processing these different sensing modalities.

In the medical field, HMIs are introduced in rehabilitation and assistive technologies. Rehabilitative technologies aim to restore impaired motor function in individuals with motor disabilities (within the limits of each individual’s disability) in order to gradually enable re-participation in activities of daily living, whereas assistive technologies attempt to allow an individual with motor disabilities to perform motor functions that are beyond their motor capabilities. In medical HMIs, bio-potentials such as electroencephalogram (EEG) [3], electromyogram (EMG) [4,5], and electroocullogram (EOG) [6]—generated by electrical sources within the human body and thus reflecting the function of organs by means of electrical activity—have been extensively utilized. Furthermore, there is a ongoing interest in signals that monitor gross muscle motion, such as force myography (FMG) [7] and mechanomyography (MMG) [8], and muscle-tendon movement such as electrical impedance tomography (EIT) [9] and medical ultrasound [10]. Hybrid HMIs have also been developed [11,12,13], which exploit complementary information of different sensing modalities and thus allow for improved control but come at the expense of increasing the complexity of the HMI.

Regarding all biological signals, the most widely employed sensing modality is surface-electromyography (sEMG) signals, a bio-potential that directly measures the electrical activity generated during voluntary contraction, since they can be easily acquired non-invasively and provide an intuitive control strategy to reproduce the function of a biological limb [2]. On the other hand, Ultrasound-Based (US-based) HMIs remain vastly unexplored compared to their sEMG counterparts, despite ultrasound sensing techniques providing a non-invasive framework for monitoring deep tissues inside the human body with high temporal resolution and sub-millimeter precision. In the context of US-based HMIs, two sensing modalities are commonly employed, namely, B-mode ultrasound and A-mode ultrasound [10]. Both modalities utilize a device, a transducer made from piezoelectric crystals that is capable of both transmitting and receiving US waves [14]. In B-mode ultrasound, phased-array transducers are utilized in order to synthesize a 2D image of the human tissues through either the combination of simultaneous emission of acoustic beams and software beam-forming or by sweep time control of the acoustic beam; whereas, in A-mode ultrasound, the simplest US sensing technique—a single transducer element—scans a line through the human body and the received echoes are plotted as a function of depth. Currently, research interest has shifted towards A-mode ultrasound sensing as it has been demonstrated that using a set of sparsely selected scanlines instead of the full imaging array does not hinder the HMI’s performance [15,16]. The benefits of utilizing a reduced number of scanlines, such as reduced computational complexity and power consumption as well as miniaturization of the instrumentation, have motivated the proposal of novel US acquisition systems [17] and recent advancements in the development of flexible fully printed transducers targeting medical applications [18].

The aforementioned characteristics of ultrasound as a sensing modality can intuitively explain the superiority of US-based HMIs for simultaneous proportional control compared to their sEMG counterparts, as it has been shown in numerous works [19,20]. More recent works include a semi-supervised framework, featuring a Sparse Gaussian Process model and principal component analysis for operating a prosthetic device with two degrees of freedom (hand grasp and wrist rotation) [21] and a novel portable US armband system with 24 channels with a multiple receiver approach, which allowed for simultaneous and proportional control of 3-DoF and 4-DoF in an online and offline setting, respectively [22]. Except from simultaneous and proportional control, a promising application of US-based HMIs is hand gesture recognition. In [23], the authors used both a novel multi-task deep learning framework and a multi-output Gaussian process for the simultaneous estimation of wrist rotation angle and recognition of finger gestures. In a more recent work, Liu et al. [24] proposed an algorithm based on a Long Short-Term Memory framework for the recognition of handwritten digits (dynamic gesture recognition) based on A-mode ultrasound sensing.

However, there are several drawbacks that hinder the application of the aforementioned HMIs in practical applications:

1. Collecting large datasets may by feasible in laboratory conditions but remains impractical for real-life scenarios, a strict requirement for conventional ML/DL algorithms known for their data-hungry nature [25].
2. It is both cost- and time-inefficient to collect representative datasets and, thus, most datasets are well-suited only within a pre-defined context. For example, the performance of a classifier significantly drops when tested on new arm positions [26,27].
3. It is assumed that dataset samples are i.i.d. sampled from the same distribution, but in real-life scenarios, the test–time distribution quickly diverges from the distribution with which the model was initially trained. Indicative causes are muscle fatigue [28] and sensor donning and doffing [29].
4. The musculoskeletal differences between individuals hinder the interoperability of the HMI leading to only subject-specific solutions [30].

In an effort to enhance the robustness of a US-Based HMI, the authors of [31] examined the potential of adaptive learning using A-mode ultrasound sensing on mitigating the performance deterioration induced by muscle fatigue. Thus, they compared the performance between conventional adaptive ML algorithms and an adaptive convolutional neural network, in which, in contrast to the former, both the feature extractor and the classifier part of the network had adaptability capabilities. In order to compare their performance, they instructed subjects to perform 15 gestures (their selection was inspired by the prominent NinaPro Database [32], extensively used as a benchmark by the sEMG-based HMI research community) once, which corresponds to one repetition, for a total of 16 repetitions. All 16 repetitions were performed without any rest to enforce muscle fatigue. The first three repetitions were used for training, the fourth repetition was used for testing, and each one of the remaining repetitions was used as a separate testing phase. During each testing phase, the predictions as well as the embeddings of the test samples were retained for obtaining both pseudo-labels and mean class embeddings used for updating the feature extractor and classifier of the network separately. By updating their network, they were able to achieve a significant 26.79% improvement in accuracy during the late stage of muscle fatigue. In their work, the classifier is updated in order to adapt to the test–time distribution of the data, which differs from the data distribution that the classifier was initially trained on.

This gap between training and test–time distributions is encountered in various domains and is often referred as concept drift in the literature [33,34]. Regarding US-Based HMIs, one can easily construct cases where the concept drift is expected to be substantially larger than the others, i.e., multiple days of the HMI being unused, subjects with large anatomical differences, and multiple donning and doffing of the sensors. All the aforementioned factors constitute a challenging environment for the development of robust US-based HMIs. Compared to the US-based HMI research community, the sEMG-based HMI research community has made significant efforts for mitigating the aforementioned optimality gap. For example, Cote Allard et al. proposed a transfer learning (TL) framework—inspired by progressive neural networks and multi-stream adaptive batch normalization—that could take advantage of multiple small datasets, allowing models to generalize well to new subjects by utilizing a single repetition from each gesture [35]. Unfortunately, TL techniques require the user to manually annotate the data acquired for re-calibrating the HMI. A more appealing scenario is to be able to re-calibrate an HMI by using newly acquired unlabeled data. This possibility is offered by Unsupervised Domain Adaptation (UDA) algorithms that take advantage of an initial labeled dataset in order to adapt a classifier to newly unlabeled data that are sampled from a similar but different distribution.

UDA algorithms have achieved remarkable results in computer vision tasks, and notable performance enhancements have also been demonstrated in inter-session re-calibration of sEMG-based HMIs by using Adaptive Batch Normalization (AdaBN) [36] and by the incorporation of domain-adversarial training in a self re-calibrating neural network [37]. Their aforementioned success and unique advantages make them a perfect candidate for exploration in different sensing modalities. In this work, we investigate the effectiveness of a wide variety of UDA algorithms in the re-calibration process of a US-based HMI. The application of UDA in a particular domain is not a straightforward process and requires experimentation for the optimization of UDA algorithms hyperparameters, design of discriminator networks used in domain-adversarial training algorithms, and even the network architectures themselves in order to fully leverage their capabilities. For our purpose, we used the Ultra-Pro dataset [23], which, to the best of our knowledge, is the only publicly available dataset. Compared to the induced muscle fatigue study [31], the Ultra-Pro dataset provides a challenging benchmark since all gestures are performed with concurrent wrist rotation and there are no intermediate data to bridge the gap between different sessions of the same subject. For assessing the performance of the UDA algorithms, we introduce an adaptation scheme, resembling the adaptation schemes introduced in the re-calibration of sEMG-based HMIs [37], where the newly acquired data arrive at two different time frames, in a shorter and a larger one from when the original labeled data were acquired, with the latter representing a more challenging scenario. Finally, we discovered that with the appropriate initialization, several UDA algorithms are capable of enhancing the performance of a US-based HMI compared to its non re-calibrated counterpart. However, in a realistic scenario, where the input modality, the data acquisition protocols, the re-calibration period, or the pattern recognition algorithm may differ, it is more likely that the performance enhancements would be rather small or not achievable at all.

The main contributions of the paper are the following:

1. A thorough examination of unsupervised domain adaptation algorithms for the re-calibration of US-based HMIs, where extensive guidelines for optimizing their performance in the field of US-based hand gesture recognition are provided. We examine domain-adversarial algorithms such as Domain Adversarial Training of Neural Networks (DANN) and its variant, Virtual Adversarial Domain Adaptation (VADA), where cluster assumption violation is incorporated in its optimization objective, as well as the non-conservative UDA algorithm, in which source domain performance is ignored, such as decision boundary refinement training with a teacher (DIRT-T), which uses VADA as initialization, and SHOT, which is also a source-data agnostic algorithm. To the best of our knowledge, this is the first time UDA algorithms have been applied to this field.
2. A new CNN-based architecture, featuring 87.92% less parameters from the state-of-the art model for US-based simultaneous estimation of wrist rotation angle and prediction of finger gestures, suitable for a UDA setting without any performance degradation on the primary task.
3. A benchmark for performance comparison of different architectures on the Ultra-Pro dataset, simulating a realistic scenario where the HMI will need to be operable shortly after user data are collected.
4. Insights about the performance of each UDA algorithm, with DANN (a domain-adversarial training algorithm) offering a 24.99% average performance enhancement and systematically improving the HMIs performance after re-calibration for all subjects and sessions. Unfortunately, we discovered that all UDA algorithms examined are unable to fully restore the HMI’s operability, even though newly acquired data for re-calibration were obtained from different within-day sessions, rendering them inappropriate for re-calibration purposes.

The rest of the paper is organized as follows. In Section 2, we introduce our methods, which include the different domain adaptation algorithms examined as well as our performance comparison benchmark on the Ultra-Pro dataset and the proposed adaptation scheme for the re-calibration of US-based HMIs tailored for UDA algorithms. In Section 3, we introduce our proposed CNN-based architecture and explain why modifications were deemed necessary for its suitability in a UDA task. In Section 4, we describe our experimental setup, which includes a brief description of the Ultra-Pro dataset and training details about both single-task and multi-task settings. In Section 5, we provide our results regarding the re-calibration performance of the UDA algorithms as well as our performance comparison results of the proposed architecture with the state-of-the-art in both single- and multi-task settings. Finally, in Section 6, we conclude our work.

## 2. Methods

### 2.1. Essential Background

#### 2.1.1. Domain Adaptation

Domain Adaptation (DA) refers to the problem of leveraging knowledge from a domain with abundance of data, referred to as source domain $D_{s}$, to improve the performance of a model in a sample-scarce domain, referred to as target domain $D_{t}$. Samples drawn from the target domain could be either labeled or unlabeled, which yields to a Supervised DA (SDA) problem or an Unsupervised DA (UDA) problem, respectively, with the latter being the hardest scenario and the subject of this paper. In a re-calibration setting, UDA algorithms can be utilized through treating the first session of each subject as their initial labeled dataset (source dataset $D_{S}$) and the rest of their sessions as newly acquired unlabeled data (target dataset $D_{T}$). The ability of UDA algorithms to perform re-calibration in an unsupervised manner makes them more appealing than TL techniques as they completely remove the burden of manual data annotation from the user. DA algorithms can be mainly categorized as divergence-based or domain adversarial training based. Regardless of their nature, all DA techniques rely on the theoretical background provided in the work of Ben-David et al. [38], which suggests that for effective domain adaptation to be achieved, predictions must be made based on features that are domain-indistinguishable.

#### 2.1.2. Commonly Used Notation

Due to the fact that domain adaptation is strongly related to source and target domain feature distribution alignment, it is convenient to think of the classifier as a composite of an embedding function and an embedding classifier h=g∘f. The embedding function $f_{\theta }:X\to Z$ maps the input data distribution *X* to a feature space Z, whereas the embedding classifier $g_{\theta }:Z\to C$ maps the embeddings to C, where C denotes the (K−1) simplex. Furthermore, it is common to denote with $D_{s}$ the joint distribution over input *x* and one-hot label *y*, and with $X_{s}$ the marginal input distribution. Both $D_{t}$ and $X_{t}$ can be analogously defined for the target domain.

### 2.2. Domain Adaptation Algorithms

#### 2.2.1. Conservative DA

In Conservative DA, we assume that the optimal classifier is the one that achieves a low generalization error in both the source and target domains. Domain adversarial training algorithms are based exactly on this assumption. Initially proposed in [39], domain adversarial training of Neural Networks (NN) allows to train a neural network to jointly learn domain-indistinguishable features that also maximize NN’s discriminability on the main task. This behavior is achieved by adding a domain classifier head to the feature extractor part of the network $f_{\theta }$, for domain discrimination based on its embeddings, and a gradient reversal layer between the feature extractor and the domain head that reverses the gradients flowing through $f_{\theta }$, which enforces feature distribution alignment in an adversarial manner. DANN was later extended to Virtual Adversarial Domain Adaptation (VADA) [40] through the incorporation of cluster violation assumption in a UDA setting. In DANN, the goal is to minimize the following objective:

(1) $$\begin{array}{c} \min_{\theta }L_{y}\left(\theta ;D_{s}\right)+\lambda_{d}L_{d}\left(\theta ;D_{s},D_{t}\right) \end{array}$$

where $L_{y}$ and $L_{d}$ in Equation (1) represent the classification and the domain loss, respectively. DANN’s inability to achieve robust and reliable adaptation under several conditions led to the incorporation of constraints that rely on the cluster violation assumption in its optimization objective [40]. Cluster violation assumption states that the input data distribution *X* contains clusters and that samples belonging to the same cluster also belong to the same class. Thus, the decision boundaries of the classifier should not cross data-dense regions. This behavior can be achieved via the incorporation of conditional entropy:

(2) $$\begin{array}{c} L_{c}\left(\theta ,D_{t}\right)=-E_{x∼D_{t}}\left[h_{\theta }\left(x\right)\log\left(h_{\theta }\left(x\right)\right)\right] \end{array}$$

which forces the classifier to be confident about its predictions and, thus, pushes the decision boundaries of the classifier further away from the target data. However, the approximation of Equation (2) breaks if the classifier is not locally-Lipschitz, which means that the classifier is allowed to abruptly change its predictions in the vicinity of the training samples. Thus, the authors decided to explicitly incorporate the locally-Lipschitz constraint via Virtual Adversarial Training (VAT) [41] by including the following additional term to the objective function:

(3) $$\begin{array}{c} L_{v}\left(\theta ,D\right)=E_{x∼D}\left[\max_{||r||<\epsilon }D_{\mathrm{KL}}(h_{\theta }\left(x\right)||h_{\theta }\left(r+\epsilon \right))\right] \end{array}$$

which enforces the classifier to be consistent about its predictions inside the norm-ball neighborhood of ϵ. VAT loss can be seen as a form of regularization; thus, it can be applied in both the source and the target domains. The combination of domain adversarial training with the semi-supervised objectives introduced as additional constraints in order to incorporate the cluster violation assumption in a domain-adversarial training setting yields the following optimization objective:

(4) $$\begin{array}{c} \min_{\theta }L_{y}\left(\theta ;D_{s}\right)+\lambda_{d}L_{d}\left(\theta ;D_{s},D_{t}\right)+\lambda_{s}L_{v}\left(\theta ;D_{s}\right)\\ +\lambda_{t}\left[L_{c}\left(\theta ;D_{t}\right)+L_{v}\left(\theta ;D_{t}\right)\right] \end{array}$$

commonly referred to as VADA. The terms $\lambda_{d}$, $\lambda_{s}$, and $\lambda_{t}$ are hyperparameters and state-of-the-art performance has been achieved across a wide variety of tasks by setting their values to $10^{-2}$, 1, and $10^{-2}$, respectively.

#### 2.2.2. Non-Conservative DA

In non-conservative DA, we assume that the optimal classifier in the target domain does not coincide with the classifier that achieves a low generalization error in both the source and the target domain. The authors of VADA [40] assume that the optimal classifier still violates the cluster assumption in the target domain and that this is what causes the aforementioned optimality gap. Their suggestion is to use VADA as initialization and further minimize the cluster assumption in the target domain. In order to make the optimization process parameterization invariant, the optimization is performed subject to the constraint of the Kullback–Leibler (KL) divergence between $h_{\theta }$ and $h_{\theta +\Delta \theta }$ being small for $x∼D_{t}$, which yields the corresponding Lagrangian:

(5) $$\begin{array}{c} \min_{\theta }\lambda_{t}\left[L_{t}\left(\theta_{n}\right)\right]+\beta_{t}E_{x∼D_{t}}\left[D_{\mathrm{KL}}(h_{\theta_{n-1}}\left(x\right)||h_{\theta_{n}}\left(x\right))\right] \end{array}$$

and can be approximated by a finite number of stochastic gradient steps. The number of refinement steps is referred to as refinement interval B. The term $L_{t}$ is referred to as target-side cluster assumption violation loss, and it is simply the sum of conditional entropy loss and VAT loss in the target domain. The model $h_{\theta_{n-1}}$ is interpreted as the sub-optimal (teacher) model for the student model $h_{\theta_{n}}$. This optimization problem is referred to as decision-boundary iterative refinement training with a teacher (DIRT-T) and, following the authors suggestions, we use ADAM with Polyak averaging for its optimization.

#### 2.2.3. Source HypOthesis Transfer (SHOT)

A more recent work [42] suggests that robust adaptation can be achieved by using solely a model trained on the source data. They rely on the assumption that the original hypothesis learned from the source domain data is closely related to the optimal hypothesis in the target domain and, thus, should remain the same $g_{s}=g_{t}$. As a result, they propose Source HypOthesis Transfer, which attempts to learn the target-specific feature extractor module via minimizing the following objective:

(6) $$\begin{array}{c} L\left(f_{t};X_{t},{\hat{y}}_{t}\right)=L_{ent}\left(f_{t};X_{t}\right)+L_{div}\left(f_{t};X_{t}\right)\\ -\beta E_{\left(x_{t},{\hat{y}}_{t}\right)∼X_{t}\times {\hat{Y}}_{t}}\sum_{k=1}^{K}I_{\left[k={\hat{y}}_{t}\right]}\log\left(h_{\theta }\left(x\right)\left[k\right]\right) \end{array}$$

The first two terms combined form the Information Maximization (IM) Loss; the first term is the conditional entropy, whereas the second term is referred to as the fair-diversity objective $L_{div}$, which enforces the classifier to predict all classes in the target domain with equal probability:

(7) $$\begin{array}{cc} L_{div}\left(h_{\theta };X_{t}\right) & =\sum_{k=1}^{K}{\hat{p}}_{k}\log{\hat{p}}_{k} \end{array}$$

(8) $$\begin{array}{cc} & =D_{\mathrm{KL}}\left(\hat{p},\frac{1}{K}1_{K}\right)-\log K \end{array}$$

where $\hat{p}=E_{x_{t}\in X_{t}}\left[h_{\theta }\left(x\right)\right]$ is the mean output embedding vector. The final term (Equation (6)) introduces a self-supervised objective, and stems from the observation that the IM Loss has the tendency to assign samples that result in low-confidence classifier predictions to the wrong clusters. At the beginning of each epoch, class prototype embeddings are computed and each sample is assigned with a pseudo-label based on the minimal distance of its embedding from the class prototypes. Class prototypes are used for generating pseudo-labels instead of the classifier predictions as they can be noisy due to domain shift. Prototype embeddings are computed from the classifier’s prediction, but in the following epochs, they are updated using the pseudo-labels obtained in the previous epoch in order to obtain new pseudo-labels. In this work, pseudo-labels are updated for five epochs but, according to the authors of SHOT, sufficiently good results can be obtained by updating the pseudo-labels once.

### 2.3. Performance Comparison Benchmark

In order to allow for a fair performance comparison between our proposed architecture and the current state-of-the-art (as implemented by us), we propose a benchmark. The motivation behind providing results of our own implementation of the current state-of-the-art is due to the limited training details provided in the respective work, which hinders the application of a standardized benchmark. To begin with, in order to allow for a fair comparison, we performed the same train–validation–test split and also treated each session separately, following the original paper [23].

We also considered it crucial to train multiple models for each experiment, as conclusions drawn from a single trial can rather be pessimistic or optimistic. Thus, for each architecture and task in hand, 10 different models were trained for each session of the Ultra-Pro dataset using different random seeds. The seeds were sampled from a uniform distribution with values ranging from 0 to $2^{32}-1$ and were used for initializing python libraries in order to make the data shuffling and weight initialization processes deterministic. The selected range captures all possible joint initialization values of the random number generations of the utilized libraries, and a uniform distribution was used for their sampling in order to avoid any bias. Since the seed number does not hold any physical meaning, the selection of a uniform distribution is the most suitable choice. Finally, by using optimal learning rates discovered for each architecture and task (see Appendix A), determined using the validation datasets of the Ultra-Pro dataset, we followed the procedure described below for comparing their task performance in each subject and session of the Ultra-Pro dataset:

1. Tasks: repeat for each task (three in total) in hand.
2. Architecture: repeat for each architecture.
3. Training details: Train 10 different models for each session of the Ultra-Pro dataset by drawing seeds from the uniform distribution described previously in this section. Each model is trained for 20 epochs using the following:
  (a) A batch size of 16;
  (b) ADAM as the optimizer;
  (c) The predetermined learning rates (see Appendix A for details).
4. Evaluation:
  - Each trained model is tested on the corresponding test set.
  - Results are reported using the mean of the performance metric (depending on the task) with standard deviation.

**Figure 1:**
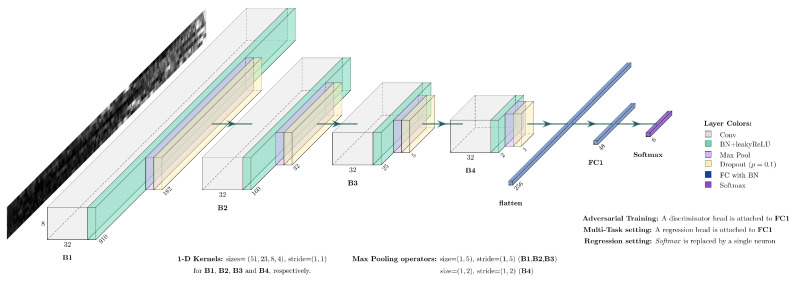
Our proposed CNN architecture, consisting of 4 distinct blocks, a fully connected layer, and a Softmax layer. Each block consists of a convolutional layer, followed by a pre-activation batch normalization layer; a max pooling layer; and, finally, a regular dropout layer with a forget rate of p=0.1. For each setting, modifications were made only after the last fully connected layer of the network. Finally, leaky ReLU with a negative slope of 0.1 was used as the activation function.

In this work, we are also interested in a performance comparison of the two architectures in a multi-task setting, where a single model simultaneously infers the finger gesture label as well as the wrist rotation angle. However, the proposed benchmark is not directly applicable to the multi-task setting. Thus, in order to apply our benchmark to the multi-task setting, we had to perform the following modifications to the overall procedure:

1. Architecture: Repeat for both architectures.
2. Multi-Task: Utilize both the class labels $y_{c}$ as well as the ground truth regression values $y_{r}$ to train the architecture using the improved automatic weighting function. For this part, a single neuron is connected in parallel to the classification head of the network (see Figure 1) for the prediction of the wrist rotation angles.
3. Training details: Train 10 different models by sampling random seeds from the uniform distribution described previously in this section. Each model is trained for twenty epochs using the following:
  (a) A batch size of 16;
  (b) ADAM as the optimizer;
  (c) The predetermined learning rates for the multi-task models (see Appendix A).
  (d) Evaluation:
    - Each model is tested in the corresponding test set. It is important to note that in the multi-task setting, a model outputs both the gesture label and the wrist rotation angle during inference.
    - Results are reported using the mean of the performance metric with standard deviation.

This standardized benchmark allows us to effectively compare the performance of different architectures on the Ultra-Pro dataset without making use of the validation sets and, thus, simulate a more realistic scenario while also avoiding any bias. However, it is important to note that the validation sets were utilized for determining the optimal learning rates for each architecture and task in hand. Furthermore, we also provide the optimal learning rates for the current state-of-the-art model, which are not provided in the respective paper [23]. Regarding the performance metrics of each task, we used *classification accuracy* for evaluating the model’s performance on the task of finger gesture recognition and the *coefficient of determination* $R^{2}$ for evaluating the model’s performance on the task of wrist rotation angle estimation. The selection of the aforementioned metrics was based on their use in relevant existing works [21,22,23].

### 2.4. Adaptation Scheme

As mentioned previously in Section 4.1, the Ultra-Pro dataset features three separate sessions for each subject. Between consecutive sessions, the subjects were allowed to rest for a reasonable amount of time, although not pre-determined as examining the robustness of the HMI across multiple within-day sessions was not on the dataset’s creators’ initial intentions, in order to prevent muscle fatigue. In this work, in order to assess the performance of UDA algorithms for the re-calibration of US-based HMIs, we introduce the following adaptation scheme. First and foremost, we treat the first session of each subject (only training data with labels) as its source dataset $D_{S1}=D_{s}$ and then treat the rest of the subject’s sessions (only training data) as our newly acquired unlabeled data used to re-calibrate the US-based HMI. This scheme allows us to create two adaptation scenarios for the re-calibration of the HMI. In our first scenario, we attempt to re-calibrate the HMI using data solely from the second session, whereas in our second scenario we attempt to re-calibrate the HMI by using data solely from the third session. The latter represents a more challenging scenario, since the time interval between the acquisition of the first and the third session is larger and will consequently result in a larger domain shift.

This particular adaptation scheme will allow us to understand to what extent the UDA algorithms could improve the performance of the HMI based on the time interval between the moment the original labeled dataset was collected and the moment the acquisition of newly unlabeled data for re-calibration occurred. Due to the within-day data acquisition of all sessions, we will not be able to provide insights about the effect of UDA on the re-calibration of an HMI for long-term enhancements, as previously demonstrated in sEMG-based research [37]. However, the proposed adaptation scheme is sufficient for obtaining insights about their performance enhancement capabilities and understand if more research effort would be worthwhile. Finally, in order to establish a baseline performance, i.e., when no unlabeled data are available for re-calibration, we used the trained CNN-based modules (with the details provided in Section 4.3) of each subject’s first session, i.e., our source dataset, and evaluated their performance on the rest of each subject’s sessions. Thus, the results of the non re-calibrated HMIs on sessions 2 and 3 will be referred to as non re-calibration results.

## 3. Proposed CNN Architecture

In this work, a new CNN architecture is proposed for the task of simultaneous wrist rotation angle estimation and finger gesture prediction as well as for each task individually. The CNN architecture for the classification task is illustrated in Figure 1. The architecture consists of four distinct blocks, a fully connected layer (acting as a bottleneck), and a *Softmax* layer. Furthermore, each block B consists of a convolutional layer, followed by a pre-activation batch normalization layer [43], a max pooling layer, and a dropout layer with a forget rate of p=0.1 [44]. It is important to mention that pre-activation batch normalization is also included in the fully connected layer of the network. Finally, we used *Leaky ReLU* with a negative slope of 0.1 as the activation function [45].

Following the work of [23], we also apply 1-D kernels, operating along the width dimension, with a stride equal to the size of the kernel in the width dimension and a stride of 1 in the height dimension. The sizes of the kernels for the convolutional layers of blocks B1, B2, B3, and B4 are 51, 23, 8, and 4, respectively. Similarly to all other DNN architectures, the trainable parameters of our network are learned in the training process by updating them using gradient-based methods and backpropagation of error algorithm. The error is associated with a loss function, which is estimated multiple times during training from a reduced subset of the initial dataset, referred to as the batch, instead of the whole dataset in order to accelerate the training process. Finally, the addition of batch normalization layers, whose transformations rely on batch statistics, allows faster convergence of our network to desirable weights for a wider range of learning rates.

**Figure 2:**
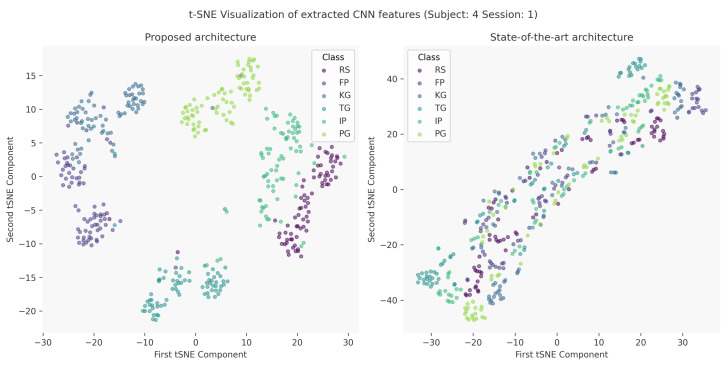
t-SNE visualization of the features learned from our network and the current state-of-the-art. Our network adopts class-discriminative features, which help alleviate the domain shift in a UDA setting, in contrast with the state-of-the-art model, which projects the samples to an 8192-dimensional feature space and adopts more generic features. Thus, the state-of-the-art model is more prone to mixing different class labels when learning domain-indistinguishable features in a UDA setting.

The motivation behind the proposal of a new architecture is twofold; first and foremost, we believe that by adopting design trends from models utilized in a UDA setting we will encourage the formation of class-discriminative clusters and thus facilitate source- and target-distribution alignment [42]. The need of class-discriminative features is highlighted by the fact that discrepancy between the two domains can be minimized by simply mixing samples from different classes, which will inevitably lead to degradation of the classification performance [46]. The trends that we adopted in our network were the use of *Leaky ReLU* as well as dropout layers in the feature extractor part of the network, which are commonly used in network architectures regarding UDA in various fields [37,40]. The proposed architecture’s ability to learn class-discriminative features is illustrated in Figure 2. Lastly, we believe that by enhancing the model’s discriminative ability on the main task, i.e., the classification of finger gestures, we will also be able to achieve better adaptation results.

### 3.1. Improving the Original CNN Model

Our experimentation with the original CNN model [23] allowed us to identify two main setbacks on its design. First and foremost, the original network is characterized by a small receptive field, which influences the quality of the extracted spatial features, and secondly, the connections from the extracted spatial features to the first fully connected layer contribute most of the network’s learnable parameters. The dimensionality of the original feature space is equal to 8192 (32 filters × 8 height × 32 width), and the first fully connected layer—in which neurons are trained to distinguish patterns of the extracted spatial features—features 48 neurons. Each neuron in the fully connected layer is connected to each individual feature and their importance is expressed through a corresponding weight. Additionally, each neuron in the first fully connected layer also includes a bias term. This results in a total of 393.264 learnable parameters (8192 features × 48 neurons plus the corresponding 48 bias terms).

Based on our observations, we decided to experiment with the depth of the network. This design choice was based on the fact that it is well known that deeper convolutional neural network architectures are capable of capturing rich and more complex features of the data [47]. Increasing the depth of the network was also deemed beneficial due to the high resolution of our input data in the width dimension. Furthermore, by increasing the depth of the network, we also managed to address the second setback we identified, as the total number of learnable parameters in the first fully connected layer is significantly reduced since the dimensionality of the proposed model’s feature space (a 256-dimensional feature space compared to the 8192-dimensional feature space of the original model) is significantly reduced. By increasing the depth of the network and experimenting with different sizes of kernels and max pooling operators, we determined the appropriate depth and layer parameters for our network by training all the different models on the sessions of the Ultra-Pro dataset and evaluating their performance on the corresponding validation sets. Finally, the derivation of the proposed model was finalized by the incorporation of the aforementioned design techniques commonly employed in a UDA setting.

### 3.2. Computational and Time Complexity Analysis

The growing interest of DNN-based solutions is mainly due to two reasons: (a) advancements made in hardware, which allow us to train and deploy, and (b) the availability of data. However, recent research efforts have focused on deriving optimal architectures under user-defined constraints and, furthermore, deploying them in resource-constrained devices such as mobile phones, drones, and robots [48,49,50]. Thus, we provide results for commonly used metrics such as the number of floating point operations, multiply–accumulate operations, and direct memory accesses to evaluate its computational efficiency as well as the corresponding memory requirements for deployment (see Table 1). Furthermore, we also provide the same results for the original model.

According to [48], our proposed CNN architecture is suitable for deployment in resource-constrained devices since the number of floating point operations required for a forward pass lies within the range of 10–150 MFLOPs. Furthermore, the inference time of 0.58 ms on our GPU and 1.21 ms on our CPU is suitable for the targeted applications since latency for prosthetic control is suggested to be within the range of 100 to 250 ms [51]. With our modifications, we are able to significantly reduce the model’s size, though at the cost of increasing the number of floating-point operations, multiply–accumulate operations, and direct memory accesses.

### 3.3. Real-World Applications of the Proposed CNN Architecture

Based on our computational complexity and memory resources analysis, our proposed architecture is rendered appropriate for applications targeting recourse-constrained devices. Thus, the possibility of potential integration to a prosthetic socket or a wearable armband is offered. The proposed CNN architecture could be integrated in an assistive technology, by assisting amputees through transcribing their indented motions to commands and control signals for operating a prosthetic arm, and in a rehabilitative technology, by enabling people with motor disabilities (e.g., neuromuscular disorders) to gradually restore motor functions within the limits of their disabilities through interactive means such as exergaming.

## 4. Experimental Setup

### 4.1. Ultra-Pro Dataset

The ultrasound-based adaptive prosthetic control (Ultra-Pro) dataset [23], is a publicly-available dataset that features US RF data as well as sEMG and Inertial Measurement Unit sensor data from the upper limb of four subjects (all transradial amputees), targeting the task of simultaneous estimation of the wrist rotation angle and recognition of finger gestures. The US RF data were captured using a customized armband, featuring eight evenly spaced transducers operating at 5 MHz, driven by a customized ultrasound system [17]. Each subject participated in three separate sessions. In each session, the subjects were instructed to perform six finger gestures—namely, Rest (RS), Fine Pinch (FP), Key Grip (KG), Tripod Grid (TG), Index Point (IP), and Power Grip (PG)—and each gesture was performed for 50 s with concurrent wrist rotation at a frequency of approximately 0.5 Hz. During the execution of the instructed movements, the data streams generated from all sensors were synchronously captured using custom specialized software. For a detailed description of the dataset, we encourage readers to refer to the original paper [23].

### 4.2. Data Prepossessing

For preprocessing the RF US signals, we followed the standard procedure of time–gain compensation, bandpass filtering, envelope detection, and log compression, adopting the procedure and the hyperparameters provided in [52] with the exception of replacing the commonly used Gaussian filter with a 101-tap bandpass FIR filter designed using the window method (hamming window). The filter is characterized by a low and a high cutoff frequency of 2 and 8 MHz, respectively. Following the work of [23], we used the processed ultrasound signals as the input modality to all networks.

### 4.3. Training Details

For hand-motion recognition, i.e., a classification task, we compared the performance of categorical cross-entropy loss and its variant with label smoothing [53]. For wrist rotation angle estimation—i.e., a regression task—mean squared error loss was used. Furthermore, we acknowledge the tedious process of determining optimal weights for each task in-hand in a multi-task learning setting and, thus, we automatically update their weights by using the loss function proposed in [54] for simultaneous hand motion recognition and wrist rotation angle prediction. The selected loss function also considers the homoscedastic uncertainty of each task [55], but the regularization term of each task τ is modified to $R_{\tau }=\ln\left(1+\sigma_{\tau }^{2}\right)$. The updated term enforces positive regularization values and, as a consequence, prevents loss from becoming negative during training.

## 5. Results

We considered it crucial to determine a fixed set of hyperparameters that can ensure near-optimal performance across subjects and sessions in order to simulate a realistic setting, where the HMI will need to be operable shortly after user data are collected. The optimal hyperparameters were determined using Optuna [56] for each combination of architectures (both ours and current state-of-the-art architectures [23]) and tasks in hand (see Appendix A).

### 5.1. Results on Single-Task Models

**Figure 3:**
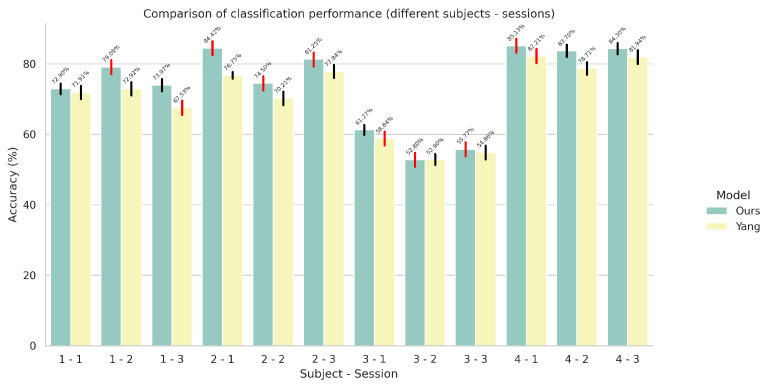
Comparison of finger gesture recognition performance in terms of accuracy between the proposed CNN-based model and the model proposed by Yang et al. [23] in a single-task setting. The red colored error bars represent standard deviations that exceed 2%.

**Figure 4:**
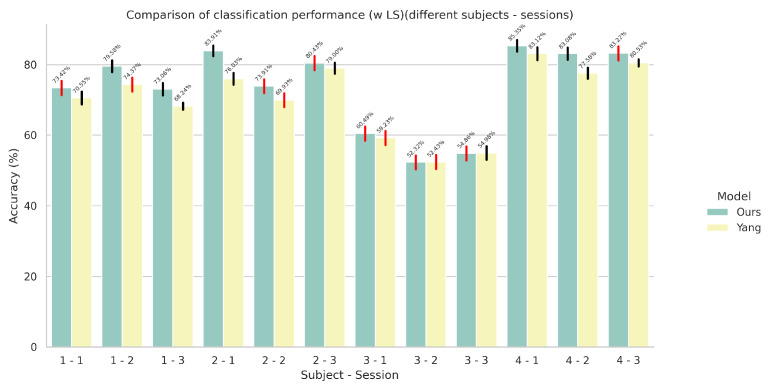
Comparison of finger gesture recognition performance (with label smoothing) in terms of accuracy between the proposed CNN-based model and the model proposed by Yang et al. [23] in a single-task setting. The red colored error bars represent standard deviations that exceed 2%.

**Figure 5:**
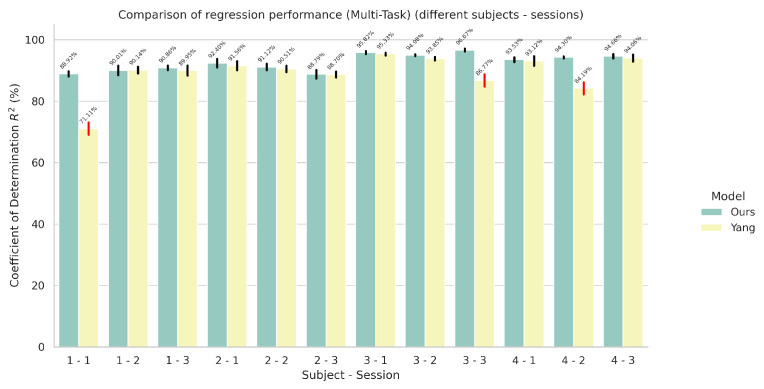
Comparison of wrist rotation angle estimation performance in terms of coefficient of determination $R^{2}$ between the proposed CNN-based model the model proposed by Yang et al. [23] in a single-task setting. The red colored error bars represent standard deviations that exceed 2%.

In this set of experiments, we compared the performance between our proposed architecture and the current state-of-the-art (our implementation) across all subjects and sessions of the Ultra-Pro dataset on the tasks of wrist rotation angle estimation and finger gesture recognition individually. For the performance comparison, we used the benchmark we proposed in Section 2.3. The results for finger gesture recognition (using cross entropy and its variant with label smoothing as the loss function) and wrist rotation angle estimation can be seen in Figure 3, Figure 4, and Figure 5, respectively.

According to our results, it is evident that our modifications to the original network, which were deemed necessary in order to improve its performance in the UDA setting, do not hinder its performance in either of the two tasks. In some cases—for example, the first session of the second subject—we notice a 7.88% overall improvement in the task of finger gesture recognition. However, we have noticed cases where the performance of our model is inferior, for example, the second session of the third subject.

### 5.2. Results on Multi-Task Models

**Figure 6:**
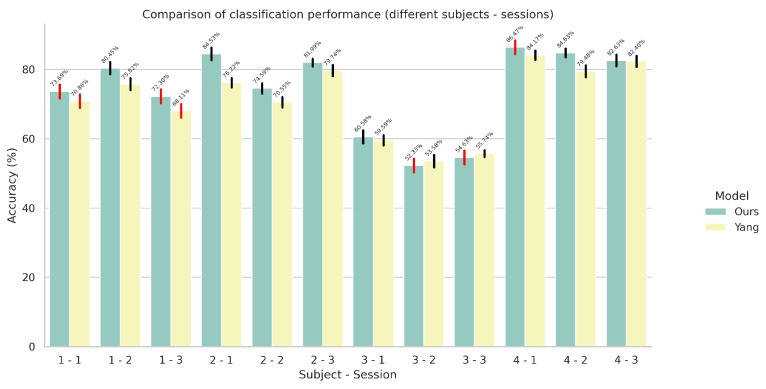
Comparison of finger gesture recognition performance in terms of accuracy between the proposed network and the network proposed by Yang et al. [23] in a multi-task setting using the improved automatic weighting loss function. The red colored error bars represent standard deviations that exceed 2%.

**Figure 7:**
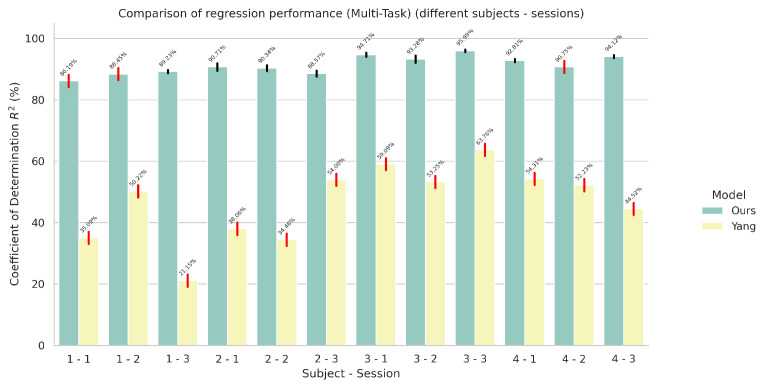
Comparison of wrist rotation angle performance in terms of coefficient of determination $R^{2}$ between the proposed network and the network proposed by Yang et al. [23] in a multi-task setting using the improved automatic weighting loss function. The red colored error bars represent standard deviations that exceed 2%.

In this set of experiments, we evaluated the performance of our proposed architecture for the task of simultaneous wrist rotation angle prediction and finger gesture classification. The results for simultaneous finger gesture recognition and wrist rotation angle estimation for the multi-task variants can be seen in Figure 6 and Figure 7, respectively.

As we can see in Figure 7, there is a substantial difference between the performance of our model compared to the performance of the original model on the wrist rotation angle estimation when using the improved loss function for automatic weighting of each task. These results indicate the need for an additional hyperparameter γ, introduced in the original paper [23], included in the automatic weighting loss function for favoring the regression task (wrist rotation angle estimation) over the classification task. The need for a hyperparameter violates the whole purpose of using an automatically weighed loss function. Instead, our model is able to achieve a better balance between the two tasks without the need of any additional hyperparameter.

### 5.3. Results on UDA

In the last set of our experiments, we evaluated the performance of all different domain adaptation algorithms on the two adaptation scenarios, introduced in Section 2.4. We have established our baselines for the non re-calibration cases, i.e., where no newly acquired data are available for re-calibration of the HMI, by inferring the best first-session models from our single-task experiments to the test dataset of the rest of each subject’s sessions. For the experimentation phase, we used the validation sets of the corresponding adaptation scenarios and monitored each individual loss term as well as the validation accuracy in order to evaluate different configurations and hyperparameters for the UDA algorithms. This was deemed crucial due to the UDA algorithm’s sensitivity to initialization. During our experimentation with domain-adversarial-based algorithms, i.e., DANN and VADA, we derived three important conclusions:

1. We found that the default hyperparameters, suggested by the corresponding authors, were the optimal hyperparameters for our case (See Appendix A). Furthermore, after several experiments with mini-batch gradient descent and its variants, we discovered that the recommended optimization algorithms with our pre-determined learning rates (for the single-task models) yielded the best results.
2. The process of jointly learning domain-indistinguishable features from both source and target domains was benefited by the use of complex discriminator networks. Through experimentation with different domain discriminator networks, we discovered that the optimal discriminator network for re-calibration of a US-based HMI featured three hidden layers, with 32, 24, and 16 neurons each.
3. We found that considering domain adversarial training with what is traditionally known as the feature extractor part of the network in CNN-based architectures, i.e., the network’s layers before the fully connected layers, leads to sub-optimal results and it is beneficial to perform domain adversarial training in order to enforce feature distribution alignment in the first fully connected layer of our network (see Figure 1). The selection of the first fully connected layer as the feature space yielded a 48-dimensional space. The consideration of a lower-dimensional feature space can also be observed in the experimental setup of SHOT [42].

For our SHOT experiments, we selected the first-session best models trained using label smoothing from our single-task experiments for initialization, as recommended by the authors of SHOT [42]. Furthermore, we discovered that the optimal learning rates used for learning the target-specific feature extractor module coincide with the learning rates used to train the initialization modules. Moreover, we followed the authors’ suggestions and adopted mini-batch gradient descent with a Nesterov momentum of 0.9 and a weight decay of $10^{-3}$ for updating the weights using backpropagation. At this point, it is important to note that due to our concerns about the optimization objective of SHOT, we considered two different cases. In the first case, we treat the SHOT optimization objective as the sum of information maximization loss and a self-supervised objective, whereas in the second case, we treat each term of the SHOT’s objective function independently, since they can represent self-sufficient objectives (for hyperparameters and details, see Appendix A).

In our last set of experiments, we decided to include the normalized first principal components of the source and target’s domains samples as supervisory signals in the optimization objective of DANN. The selection of the normalized principal component as a supervisory signal was based on the fact that it is linearly related to the wrist rotation angle and can also be obtained easily in an unsupervised manner [57]. Our motivation was to investigate if the performance of DANN could be augmented by the incorporation of two auxiliary tasks, i.e., estimating the wrist rotation angle in both the source and the target domains. The incorporation of the two auxiliary tasks in DANN yields the following optimization objective:

(9) $$\begin{array}{c} \min_{\theta }L_{y}\left(\theta ;D_{s}\right)+\lambda_{r}\left[L_{r-source}\left(\theta ;D_{s}\right)+L_{r-target}\left(\theta ;D_{t}\right)\right]+\lambda_{d}L_{d}\left(\theta ;D_{s},D_{t}\right) \end{array}$$

where $\lambda_{r}$ is a hyperparameter for controlling the influence of the two regression objectives during domain-adversarial training, which was set to 0.35. We also performed experiments using the ground truth regression labels; in this case, the hyperparameter was set to 0.45. We will refer to the results of DANN with self-supervision and ground truth wrist rotation angles as DANN SS and DANN GT, respectively. Finally, since the normalized principal component is an approximation of the true wrist rotation angle, we used mean absolute error instead of mean squared error in order to mitigate the influence of possible outliers.

Finally, model selection for each UDA algorithm was performed by selecting the weights that minimized its corresponding training loss. The accuracy results of the different UDA algorithms are presented in Table 2.

From the obtained results, we drew the following conclusions:

1. The algorithms that rely on domain adversarial training, i.e., DANN and VADA, perform better than SHOT, which is a source-data agnostic UDA algorithm. This means that in the case where the domain shift is large, it is beneficial to jointly learn domain-indistinguishable features and maximize classifier’s discriminability on the primary task.
2. It is beneficial to use DIRT-T to further minimize target-side cluster assumption in cases where the domain shift is small—for example, both adaptation scenarios of subject 4—but does not always guarantee better results.
3. Compared to all the UDA algorithms, DANN offers the greatest performance enhancements with an average improvement of 24.99%. Unfortunately, re-calibration using SHOT can even have a negative impact on the performance of the HMI.
4. Incorporation of the wrist rotation angle as a supervisory signal in the DANN objective, either by using the ground truth values or by obtaining them in an unsupervised manner by utilizing the normalized first principal component, does not lead to better results than re-calibrating the HMI by solely using DANN.

## 6. Conclusions and Future Work

Inspired by sEMG-based HMI research, we investigated the possibility of re-calibrating US-based HMIs using unlabeled data from different within-day sessions by employing state-of-the-art UDA algorithms. This is a more challenging task than making a classifier adapt to muscle fatigue [31], where no continuous stream of data is available for continual adaptation, in a more demanding dataset featuring concurrent wrist rotation. For our experiments, we introduced an adaptation scheme on the publicly available Ultra-Pro dataset, which allows us to investigate the performance enhancements of UDA algorithms through two adaptation scenarios based on the time interval between the initial label dataset that was collected and the acquisition of unlabeled data used for re-calibration. Our experimentation also led to the proposal of a new architecture, featuring 87.92% less parameters than the current state-of-the-art. For effective performance comparison of the two architectures, we introduced a benchmark that simulates a realistic scenario while also avoiding any bias, where no validation set will be available and the HMI will need to be operable shortly after user data are collected. Furthermore, we provide extensive guidelines for the re-calibration of US-based HMIs using UDA algorithms and draw important conclusions on their drawbacks and performance. According to our findings, our proposed CNN-based architecture achieves similar performance with the current state-of-the-art [23] while featuring only 50,582 trainable parameters instead of 401,382. Also, by using DANN, a domain-adversarial based algorithm, we achieved a 24.99% average performance enhancement and systematically improved the classification performance of a US-based HMI compared to its non re-calibrated counterpart for all subjects and sessions. However, our results indicate that in cases where the data acquisition process, the re-calibration period, and the network architecture may differ and proper initialization of the UDA algorithms may not be feasible, the observed enhancements would be rather small or even not noticeable.

We believe that the findings of our work raise several research questions that need to be addressed. To begin with, UDA algorithms require careful initialization in order to fully leverage their capabilities and cannot fully restore the operability of a US-based HMI. These results indicate the need for a online learning learning algorithm, capable of adapting in dynamic environments where test–time data continuously change in unforeseen ways. Furthermore, it is important to ensure that important information will not be lost during the dynamic adaptation of the model, a common issue that is often referred to as catastrophic forgetting [58]. To continue with, most of the aforementioned techniques rely on backpropagation of error algorithm to update the network’s parameters, a non-local and computationally intensive learning rule. Based on our observations, our main research focus would be the development of an online continual learning algorithm, capable of adapting to dynamic environments using local learning rules. Finally, we plan to collect data in order to construct a representative dataset, featuring hand movements performed in activities of daily living and multiple sessions for a better evaluation of the re-calibration performance of different algorithms.

## Figures and Tables

**Table 1:** Computational efficiency and memory resources requirements for the proposed architecture.

Model	Parameters	MFLOPs	MMACs	MDMACs	Inference Time (ms)	Inference Time CPU (ms)	Memory (MB)
Proposed Model	401,382	87.77	43.97	44.51	0.58	1.21	0.193
Original model	50,582	85.34	42.64	43.62	0.25	0.191	1.598

**Table 2:** Re-calibration accuracy results of Unsupervised Domain Adaptation algorithms.

	1-1	1-2	1-3	2-1	2-2	2-3	3-1	3-2	3-3	4-1	4-2	4-3
No Re-calibration	0.740	0.238	0.264	0.879	0.291	0.202	0.684	0.291	0.349	0.482	0.482	0.336
DANN	-	0.287	0.402	-	0.395	0.247	-	0.316	0.396	-	0.580	0.428
DANN (w LS)	-	0.293	0.322	-	0.258	0.231	-	0.342	0.415	-	0.588	0.397
VADA	-	0.313	0.380	-	0.224	0.182	-	0.316	0.371	-	0.542	0.398
VADA (w LS)	-	0.266	0.376	-	0.356	0.235	-	0.337	0.415	-	0.611	0.408
DIRT-T	-	0.282	0.428	-	0.340	0.235	-	0.317	0.396	-	0.644	0.435
SHOT (original)	-	0.200	0.295	-	0.242	0,178	-	0.302	0.297	-	0.529	0.378
SHOT (ours)	-	0.213	0.311	-	0.240	0.180	-	0.307	0.303	-	0.531	0.382
DANN-GT	-	0.262	0.367	-	0.322	0.200	-	0.362	0.438	-	0.571	0.371
DANN-SS	-	0.256	0.287	-	0.407	0.238	-	0.341	0.442	-	0.351	0.442

Bold entries indicate the best re-calibration results for each adaptation scenario.

## Data Availability

The Ultra-Pro dataset used in this article is available online: https://doi.org/10.6084/m9.figshare.20448489.v1.

## Abbreviations

The following abbreviations are used in this manuscript:

HMI	Human–Machine Interface
UDA	Unsupervised Domain Adaptation
US	Ultrasound
CNN	Convolutional Neural Network
DANN	Domain-adversarial Training of Neural Networks
EEG	Electroencephalogram
EMG	Electromyogram
EOG	Electroocullogram
FMG	Force Myography
MMG	Mechanomyography
EIT	Electrical Impedance Tomography
sEMG	Surface-electromyography
ML	Machine Learning
DL	Deep Learning
NinaPro	Non-Invasive Adaptive Hand Prosthetics
TL	Transfer Learning
AdaBN	Adaptive Batch Normalization
Ultra-Pro	Ultrasound-based Adaptive Prosthetic Control
DA	Domain Adaptation
SDA	Supervised Domain Adaptation
VADA	Virtual Adversarial Domain Adaptation
ADAM	Adaptive Moment Estimation
VAT	Virtual Adversarial Training
DIRT-T	Decision Boundary Iterative Refinement Training with a Teacher
SHOT	Source Hypothesis Transfer
SGD	Stochastic Gradient Descent

## Appendix A. Hyperparameters

**Table A1:** Optimal learning rates for single-task models.

Task	Finger Gesture Recognition w/o LS	Figure Gesture Recognition w LS	Wrist Rotation Angle Estimation
Proposed model	0.0026	0.0035	0.0008
Original model	0.00011	0.00018	0.000079

**Table A2:** Optimal learning rates for multi-task models.

	Learning Rate
Proposed model	0.0014
Original model	0.00012

**Table A3:** Hyperparameters for DANN–VADA configurations.

UDA Algorithm	Domain Weight $\lambda_{d}$	Regression Weight $\lambda_{r}$	VAT Source $\lambda_{s}$	Target-Side CA Violation $\lambda_{t}$
DANN	0.1	-	-	-
DANN SS	0.1	0.36	-	-
DANN GT	0.1	0.45	-	-
VADA	0.1	-	1	$10^{-2}$

**Table A4:** Hyperparameters for SHOT.

UDA Algorithm	Information Maximization Loss	Self-Supervised Objective (β)
SHOT	1	0.1

**Table A5:** Hyperparameters for SHOT (our variant).

UDA Algorithm	Conditional Entropy	Fair Diversity	Self-Supervised Objective (β)
SHOT	1.0	0.7	0.1
